# Supplementary material for: Altered Sweat Composition Due to Changes in Tight Junction Expression of Sweat Glands in Cholinergic Urticaria Patients
Source: Int J Mol Sci. 2024 Apr 25;25(9):4658. doi: 10.3390/ijms25094658 (PMC11083780; doi:10.3390/ijms25094658)
Supplement: Supplementary file 1 [file ijms-25-04658-s001.zip › ijms-2901265-supplementary.pdf]

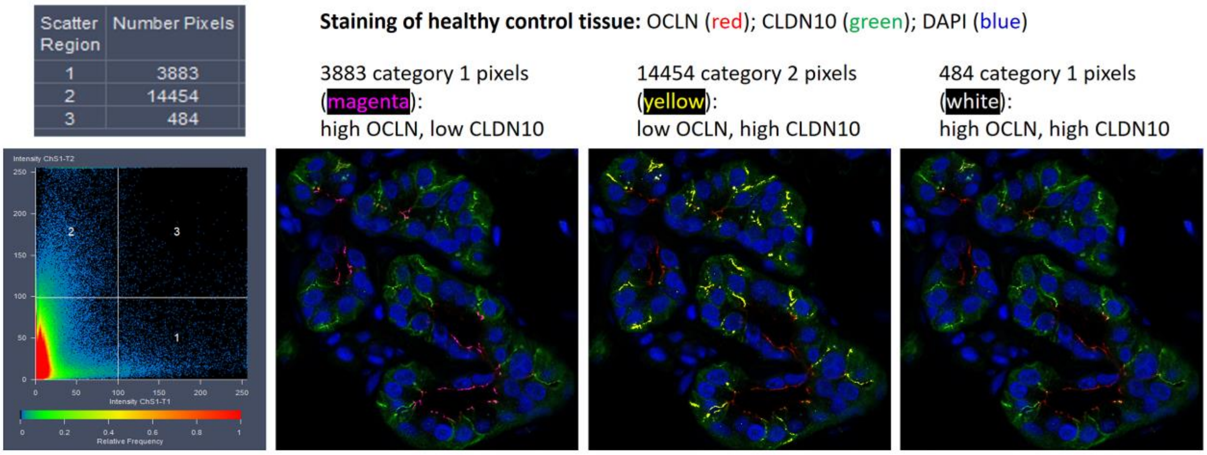

Figure S1: Colocalization analysis. As an example, a sweat gland section from a healthy control co-stained for OCLN (red), CLDN10 (green) is shown. Nuclei (DAPI, blue) were stained for better orientation. Thesholding yielded 3883 pixels with high OCLN and low CLDN10 staining (highlighted in magenta in the left image), 14454 pixels with low OCLN and high CLDN10 staining (highlighted in yellow in the middle image) and 484 pixels with high OCLN and high CLDN10 staining (highlighted in white in the right image). The percentage of OCLN signals co-localized with CLDN10 would thus be 484 / (3883+484)*100% = 11.1%.

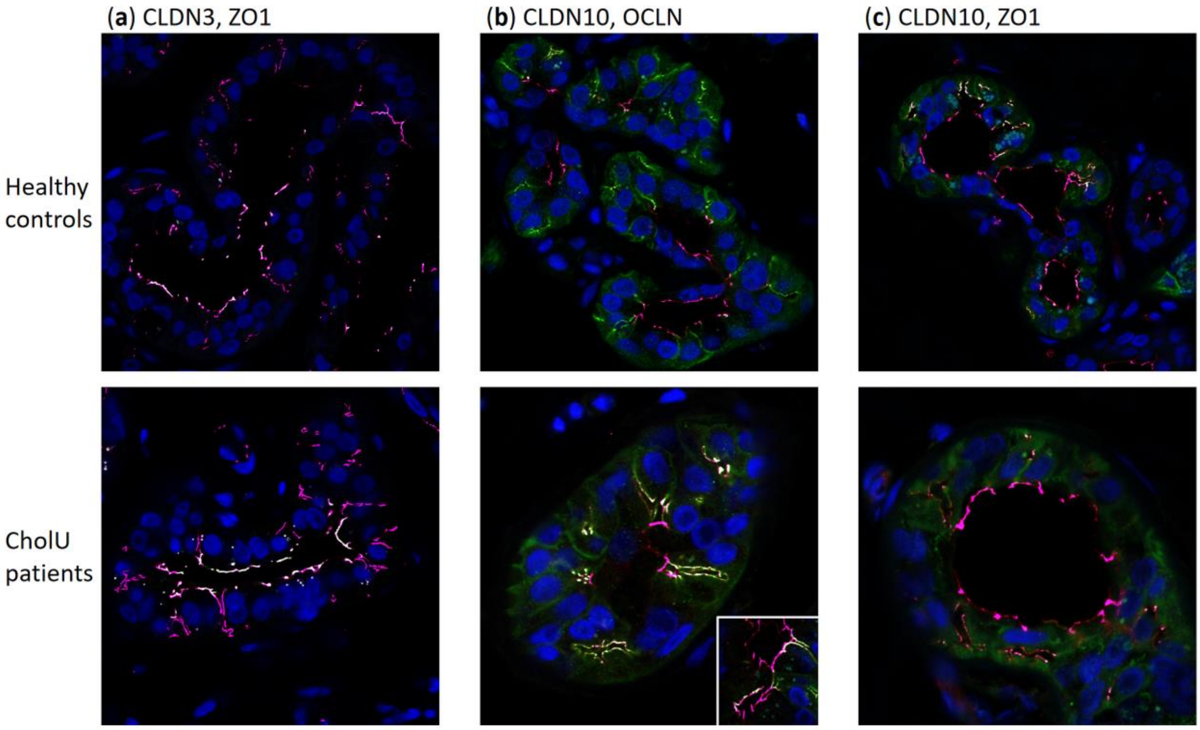

Figure S2: Examples for colocalization analyses. Analyses were carried out as explained in Figure S1. Results for different sections from one individual were averaged and the resulting mean included as one data point in Figure 2d – f. (**a**) Co-staining for CLDN3, ZO1 and DAPI. Pixels with high ZO1 and low CLDN3 staining are highlighted in magenta, pixels with high ZO1 and high CLDN3 staining in white. The percentage of ZO1 signals co-localized with CLDN3 amounts to 16.3% in the healthy control and 25.4% in the CholU patient sample. CLDN3 is restricted to the central duct in the healthy control but also found at the base of canaliculi in the CholU patient sample. (**b**) Co-staining for CLDN10, OCLN and DAPI. Pixels with high OCLN and low CLDN10 staining are highlighted in magenta, pixels with high OCLN and high CLDN10 staining in white. In the healthy control CLDN10 (green) is restricted to the canaliculi whereas OCLN (magenta) is restricted to the central duct with only small overlap of 11.1% (white, same sample as shown in Figure S1). In the CholU patient sample there is considerable overlap of high OCLN and CLDN10 signals (white; 48.6%) in the canaliculi, indicating redistribution of OCLN into the canaliculi. As shown in the inset, in other sections redistribution is also found for CLDN10 into the TJs of the central duct. (**c**) Co-staining for CLDN10, ZO1 and DAPI. Pixels with high ZO1 and low CLDN10 staining are highlighted in magenta, pixels with high ZO1 and high CLDN10 staining in white. The percentage of ZO1 signals co-localized with CLDN10 amounts to 37.3% in the healthy control and 2.1% in the CholU patient sample. As in (b), CLDN10 is predominantly found in the canaliculi in the healthy control. In the CholU patient sample CLDN10 is almost lost from the canaliculi.
